# Supplementary material for: Genetic variation in promoter region of the bovine LAP3 gene associated with estimated breeding values of milk production traits and clinical mastitis in dairy cattle
Source: PLoS One. 2023 May 19;18(5):e0277156. doi: 10.1371/journal.pone.0277156 (PMC10198522; doi:10.1371/journal.pone.0277156)
Supplement: S1 Table — H = Haplotypes. (DOCX) [file pone.0277156.s001.docx]

| **Haplotype** | **g.37140485C>G** | **g.37140513T>C** | **g.37140514A>G** | **g.37140644C>T** | **g.37140681G>A** | **g.37140767C>T** | **g.37140789T>G** | **Frequency** |
| --- | --- | --- | --- | --- | --- | --- | --- | --- |
| H1 | **C** | **T** | **A** | **C** | **G** | **C** | **T** | **0.4272** |
| H2 | **C** | **T** | **G** | **C** | **G** | **T** | **T** | **0.1956** |
| H3 | **G** | **C** | **G** | **T** | **A** | **C** | **G** | **0.1668** |
| H4 | **C** | **T** | **G** | **C** | **G** | **C** | **T** | **0.1484** |
| H5 | C | T | A | C | G | C | G | 0.0109 |
| H6 | G | T | G | C | G | T | T | 0.009 |
| H7 | C | T | G | T | G | T | T | 0.0071 |
| H8 | G | T | G | C | G | C | T | 0.0069 |
| H9 | G | C | G | T | A | C | T | 0.0048 |
| H10 | C | C | G | C | G | C | T | 0.0047 |
| H11 | G | T | A | C | G | C | T | 0.003 |
| H12 | G | C | G | T | A | T | G | 0.003 |
| H13 | C | T | G | T | G | C | T | 0.0024 |
| H14 | C | T | A | C | A | C | T | 0.0024 |
| H15 | G | T | G | T | G | C | T | 0.0024 |
| H16 | C | T | G | C | G | C | G | 0.001 |
