# Supplementary material for: Genetic variation in promoter region of the bovine LAP3 gene associated with estimated breeding values of milk production traits and clinical mastitis in dairy cattle
Source: PLoS One. 2023 May 19;18(5):e0277156. doi: 10.1371/journal.pone.0277156 (PMC10198522; doi:10.1371/journal.pone.0277156)
Supplement: S2 Table — a,bMeans with different superscripts are significantly different (P < 0.05); α = Allele substitution effect. (DOCX) [file pone.0277156.s002.docx]

| **Loci** | **Genotype** | **LMY (kg)** | **305dMY (kg)** |
| --- | --- | --- | --- |
| rs720373055:T>C | TT (136) | 1.26^b^ | -141.62^b^ |
|  | TC (61) | -136.50^c^ | -83.61^b^ |
|  | CC (15) | 633.08^a^ | 627.35^a^ |
|  | **P** | 0.00614 | 0.0030 |
|  | **α** | -100.07 | -225.065 |
| rs720349928:G>A | GG (140) | 401.20^a^ | 260.66^a^ |
|  | GA (59) | 288.78^ab^ | 305.14^a^ |
|  | AA (13) | -192.14^b^ | -163.69^b^ |
|  | **P** | 0.0001 | <.0001 |
|  | **α** | 272.75 | 299.45 |
