# Supplementary material for: Genetic variation in promoter region of the bovine LAP3 gene associated with estimated breeding values of milk production traits and clinical mastitis in dairy cattle
Source: PLoS One. 2023 May 19;18(5):e0277156. doi: 10.1371/journal.pone.0277156 (PMC10198522; doi:10.1371/journal.pone.0277156)
Supplement: S3 Table — ** = P < 0.01; * = P < 0.05; CI = Confidence interval; P/calving = Period of calving; S/calving = Season of calving. (DOCX) [file pone.0277156.s003.docx]

| **Effect** |  | **Wald chi square** | **Odds ratio** | **95% CI** |
| --- | --- | --- | --- | --- |
| Breed^***^ | Sahiwal | 13.802 | 6.85 | 2.48-18.89 |
|  | Karan Fries | - | - | - |
| P/calving** | 5 | 0.74 | 4.41 | 0.856-22.68 |
|  | 6 | 1.76 | 4.61 | 1.78-11.95 |
|  | 7 | - | - | - |
| S/calving* | 1 | 0.03 | 2.02 | 0.525-7.79 |
|  | 2 | 8.58 | 8.87 | 1.602-49.17 |
|  | 3 | 1.79 | 1.17 | 0.234-5.867 |
|  | 4 | - | - | - |
