# Supplementary material for: Genetic variation in promoter region of the bovine LAP3 gene associated with estimated breeding values of milk production traits and clinical mastitis in dairy cattle
Source: PLoS One. 2023 May 19;18(5):e0277156. doi: 10.1371/journal.pone.0277156 (PMC10198522; doi:10.1371/journal.pone.0277156)
Supplement: Attachment — Submitted filename: Response to Reviewers.docx [file pone.0277156.s005.docx]

**Rebuttal letter that responds to the point raised by the academic editor and reviewers to the manuscript PONE-D-22-15483R1: “Genetic variation in promoter region of the bovine LAP3 gene associated with estimated breeding values of milk production traits and clinical mastitis in dairy cattle”**

Review Comments to the Author

**Reviewer #1**: The authors have adequately addressed the raised comments; I am satisfied and recommend the manuscript accepted for publication.

**Response**: Authors are thankful for the comment.

**Reviewer #2**: All required questions have been answered and the authors have responded satisfactorily to my comments.

**Response**: Authors are thankful for the comment.

Additional minor corrections to the manuscript:

We have added the approval no. to the ethical statement at the materials and methods section of the revised manuscript (line number 128). Moreover, we have included one additional reference to the reference section of revised manuscript (line number 475).
